# Low Surface Potential with Glycoconjugates Determines Insect Cell Adhesion at Room Temperature

**DOI:** 10.1021/acs.jpclett.2c01673

**Published:** 2022-10-06

**Authors:** Takahisa Matsuzaki, Daigo Terutsuki, Shoma Sato, Kohei Ikarashi, Kohei Sato, Hidefumi Mitsuno, Ryu Okumura, Yudai Yoshimura, Shigeyoshi Usami, Yusuke Mori, Mai Fujii, Shota Takemi, Seiichiro Nakabayashi, Hiroshi Y. Yoshikawa, Ryohei Kanzaki

**Affiliations:** † Center for Future Innovation, Graduate School of Engineering, Osaka University, 2-1, Yamadaoka, Suita, Osaka 565-0871, Japan; ‡ Department of Applied Physics, Graduate School of Engineering, Osaka University, Suita, Osaka 565-0871, Japan; § Division of Strategic Research and Development, Saitama University, Shimo-Okubo 255, Sakura-Ku, Saitama 338-8570, Japan; ∥ Research Center for Advanced Science and Technology, The University of Tokyo, 4-6-1 Komaba, Meguro-Ku, Tokyo 153-8904, Japan; ⊥ Department of Finemechanics, Graduate School of Engineering, Tohoku University, 6-6-01 Aramaki-aza Aoba, Aoba-Ku, Sendai, 980-8579 Japan; # Department of Chemistry, Saitama University, Shimo-Okubo 255, Sakura-Ku, Saitama 338-8570, Japan; ¶ Graduate School of Science and Technology, Shizuoka University, 3-5-1 Johoku, Hamamatsu, Shizuoka 432-8561, Japan; □ Course of Applied Chemistry and Biochemical Engineering, Department of Engineering, Graduate School of Integrated Science and Technology, Shizuoka University, 3-5-1 Johoku, Hamamatsu, Shizuoka 432-8561, Japan; ○ Department of Applied Chemistry and Biochemical Engineering, Faculty of Engineering, Shizuoka University, Shizuoka 432-8561, Japan; △ Research Institute of Green Science and Technology, Shizuoka University, 3-5-1 Johoku, Hamamatsu, Shizuoka 432-8561, Japan; ▽ Department of Microbiology and Immunology, Graduate School of Medicine, Osaka University, Osaka 565-0871, Japan; ⬡ WPI Immunology Frontier Research Center, Osaka University, Osaka 565-0871, Japan; ■ Integrated Frontier Research for Medical Science Division, Institute for Open and Transdisciplinary Research Initiatives, Osaka University, Osaka 565-0871, Japan; ● Division of Electrical, Electronic and Info communications Engineering, Graduate School of Engineering, Osaka University, Suita, Osaka 565-0871, Japan; ▲ Area of Regulatory Biology, Division of Life Science, Graduate School of Science and Engineering, Saitama University, 255 Shimo-Okubo, Sakura-Ku, Saitama 338-8570, Japan

## Abstract

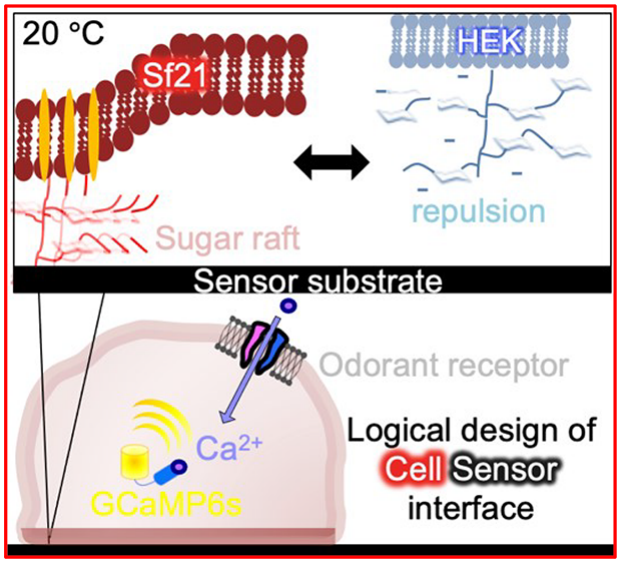

Cell-coupled field-effect transistor (FET) biosensors have attracted considerable attention because of their high sensitivity to biomolecules. The use of insect cells (Sf21) as a core sensor element is advantageous due to their stable adhesion to sensors at room temperature. Although visualization of the insect cell–substrate interface leads to logical amplification of signals, the spatiotemporal processes at the interfaces have not yet been elucidated. We quantitatively monitored the adhesion dynamics of Sf21 using interference reflection microscopy (IRM). Specific adhesion signatures with ring-like patches along the cellular periphery were detected. A combination of zeta potential measurements and lectin staining identified specific glycoconjugates with low electrostatic potentials. The ring-like structures were disrupted after cholesterol depletion, suggesting a raft domain along the cell periphery. Our results indicate dynamic and asymmetric cell adhesion is due to low electrostatic repulsion with fluidic sugar rafts. We envision the logical design of cell–sensor interfaces with an electrical model that accounts for actual adhesion interfaces.

Cell-coupled FET biosensors based on complementary metal-oxide semiconductors (CMOS) have attracted much attention owing to their high sensitivity to biomolecules and portability.^1−4^ The lateral and axial morphology between the cell and the electrode interface is believed to play an essential role in the efficiency of electrical signal transfer.^5,6^ Various optical microscopy techniques have been combined to gain deeper insight into the cell–electrode interface in biosensors. For example, fluorescence interference contrast microscopy (FLIC) has investigated the neuronal cell–substrate interface by taking advantage of its high axial resolution.^7,8^ FLIC requires the reconstruction of silica steps with different thicknesses on the sensor surface, and the topological structures themselves denature the native cell adhesion.^9^ Moreover, by transmission electron microscopy (TEM), Wrobel et al. reported that the distribution of interfacial gaps in human embryonic kidney-293T (HEK) cells and sensors ranged from areas of strong adhesion (≤10 nm) to areas of weak adhesion (10–100 nm).^10^ Repeating the cryo-dehydration process for the fixation is likely to affect the dynamics of insect cell adhesion. Therefore, microscopic techniques to visualize the interfaces between “living” cells and sensors without altering the sensor surface are necessary for the rational development of sensor devices.

In contrast, IRM exploits interference between light reflected from sample–liquid and liquid–substrate interfaces. In the case of the glass substrate used, IRM can be a prominent label-free technique for measuring the distance between cells and substrates with a high axial resolution of *z* ∼ 2 nm.^11^ Moreover, such precise visualization of the sample–substrate interface has the advantage of assessing biological interactions such as focal adhesion assembly^12,13^ and physical interaction^14,15^ such as repulsion of glycoconjugates^16^ (dense glycocalyx, composed of glycolipids, glycoproteins, etc.). Recently, we have developed “insect” cell-coupled FET devices with high selectivity for odorant molecules through the strong expression of odorant receptors (OR).^17,18^ Minor structural differences in similar silk moth pheromone components (i.e., bombykol and bombykal) were sensitively discriminated based on drain current modulations of the sensors at room temperature. Previously, a scanning electron microscope (SEM) has clearly visualized dehydrated *Spodoptera frugiperda* (Sf21) cells in close contact with a sensor surface.^19^ However, spatiotemporal insights into the adhesion dynamics of intact insect cells and their origins are still unknown. The combination of IRM with FET-device sensors should therefore provide spatiotemporal information about the cell-sensor interfaces.

In this study, we quantitatively assessed the adhesion dynamics of *Spodoptera frugiperda* (Sf21) cells to a substrate by using IRM at room temperature (20 °C) in phosphate buffered saline (PBS, calcium-free) (Schematics; Figure S1a). To compare adhesion dynamics between mammalian cells, we used human embryonic kidney (HEK) cells, which are a classical sensor element for targeted molecules in mammals.^20^ Transmission images of HEK cells showed dynamic morphological changes (i.e., elongated to spherical shape), whereas Sf21 cells remained spherical (Figure S1b). In contrast, IRM images of HEK cells showed a point of contact during incubation (i.e., small dark region surrounded by bright interference fringes), and Sf21 showed detectable interference fringes immediately after contact with the substrate (*t* ∼ 0 min), which then spread radially and formed visible dark ring structures at the cell periphery (*t* ∼ 30 min, Figure 1b middle). To quantitatively assess the cell adhesion from interference images, the contact area was cropped according to previous protocols^12,13^ (see also methods in the Supporting Information) (Figure 1b bottom). Here, by changing the intensity threshold factor, the contact area (*h* ≤ 40 nm) was optimized for evaluation of the peripheral strong adhesion zone and small patch-like structures (∼1 μm) (Figure S1c). The contact area of Sf21 is larger than that of HEK (*t* = 30 min). Statistical analysis showed that the contact area of Sf21 exhibited a sigmoidal-like drastic increase and reached a plateau at *t* ∼ 10 min, whereas that of HEK remained constant (Figure 1c). This increase in the contact area was 10 times higher than that of HEK (*t* ∼ 10 min). The difference in dynamics between insect and mammalian cells is shown schematically (Figure 1c inset). These results clearly highlight the dynamic adhesion fingerprints of insect cells compared to those of mammalian cells. The distance between cell membranes and a sensor substrate plays a crucial role in the efficiency of electrical signal transfer, and a shorter distance is beneficial for a better signal-to-noise ratio.^21^ Therefore, Sf21 cells are suitable for cell-FET devices owing to their unique adhesion properties.

**Figure 1:**
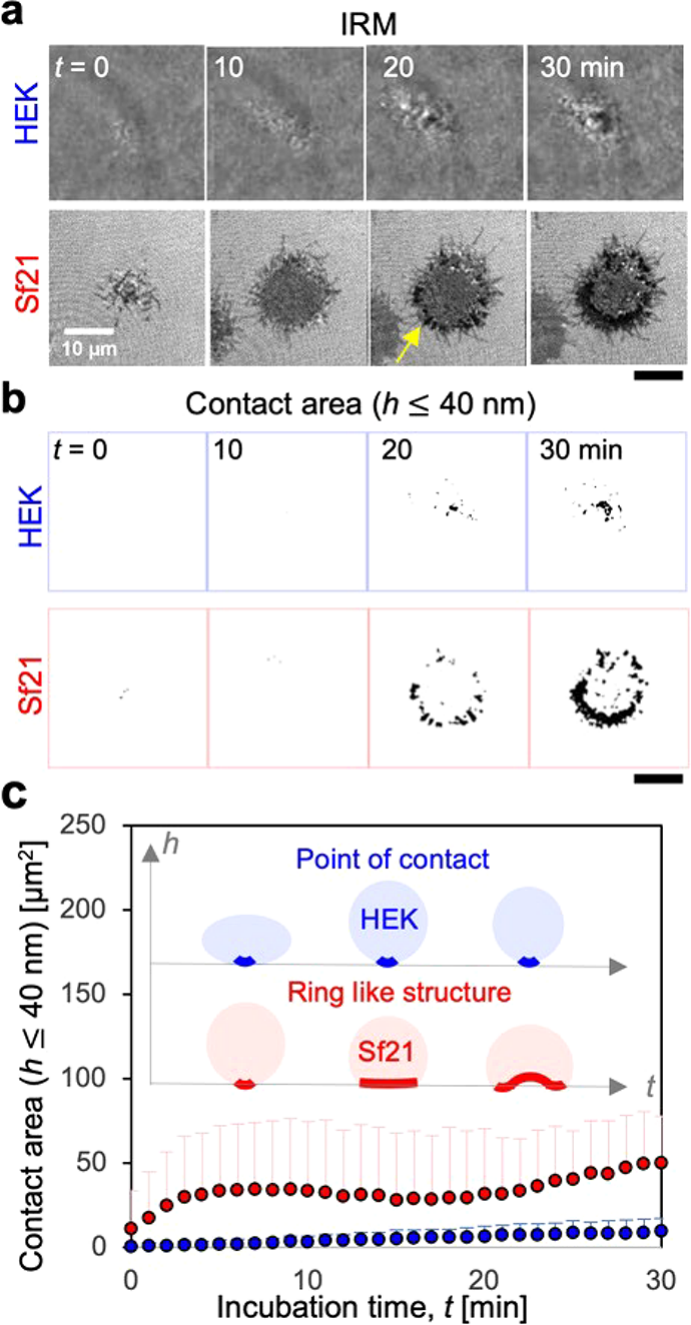
Dynamic adhesion of insect and mammalian cells at room temperature (20 °C). Time course of (a) IRM images and (b) cropped contact area (*h* ≤ 40 nm) of HEK and Sf21 cells. (c) Statistical analysis of the corresponding contact area during incubation (HEK, *n* = 6 cells; Sf21, *n* = 9 cells; two independent experiments for the analysis). The error bar represents the standard deviation. The inset shows a schematic representation of the adhesion morphology of Sf21 and HEK cells along the optical axis. The scar bar corresponds to 10 μm.

The next question is to uncover the surface molecular origins that determine the adhesion signatures of insect cells. Adhesion of mammalian cell lines is thought to be controlled by both biological and physical interactions.^12−16^ Although the suspension medium did not contain any adhesion molecules (i.e., fibronectin collagen, etc.), we confirmed that Sf21 cells did not express focal adhesion kinase in the absence of serum (Figure S2a). It should be noted that the culture medium in the presence of serum resulted in the formation of focal adhesions in both insect and mammalian cells (Figure S2b). Moreover, the representative protease trypsin did not significantly suppress the contact area (Figure S2c); thus, the biological factor is not related to the strong adhesion signatures of insect cells. To gain insight into the physical electrostatic properties of the insect cell surface, we measured the zeta potential (Figure 2a top). HEK cells showed a strong negative potential, possibly due to negative sialic glycoconjugates.^22^ Sf21 cells showed a weaker negative potential than HEK cells, leading to two scenarios: (1) the presence of a specific sialic glycoconjugate with low electrical potential or (2) low amounts of sialic glycoconjugate. To test this hypothesis, cells were treated with enzymes (α2-3,6,8 neuraminidase) that specifically degrade sialic acid. Pretreatment with the enzyme reduced the zeta potential of HEK cells to that of Sf21 cells, whereas the surface potential of Sf21 cells was intact (Figure 2a bottom). To determine the amount of glycoconjugates on the surface, fluorescent wheat germ agglutinin (WGA), which binds specifically to *N*-acetyl-d-glucosamine and sialic acid was used (Figure 2b left). The fluorescence intensity of Sf21 cells was higher than that of HEK cells, demonstrating the presence of a greater amount of glycoconjugates on the surface of insect cells (Figure 2c,d). In addition, treatment with neuraminidase significantly reduced fluorescence intensity (Figure 2b right, Figure 2c,d right). The results support scenario 1: a unique glycoconjugate of Sf21 cells based on *N*-acetyl-d-glucosamine and sialic acid with a lower electrostatic potential. Classical studies have suggested that the types of sugar components in insect cells differ from those in mammals.^23,24^ Therefore, a specific glycoconjugate content should be present on the surface. After treatment with neuraminidase, IRM detected a significantly larger area of homogeneous dark contrast (*t* ∼ 10 min) (Figure 2e). This dramatic increase in the contact area of insect cells was three times higher than that of cells without neuraminidase treatment (control, Figure 2f, *t* ∼ 10 min). Notably, the strong adhesion with ring structures (yellow arrow in Figure 1) was significantly suppressed. These results clearly indicate that the adhesion signatures of sf21 cells are controlled by surface-specific glycoconjugates with low surface potentials. Moreover, specific ring structures at the cell periphery were supported by the local assembly of the glycoconjugate.

**Figure 2:**
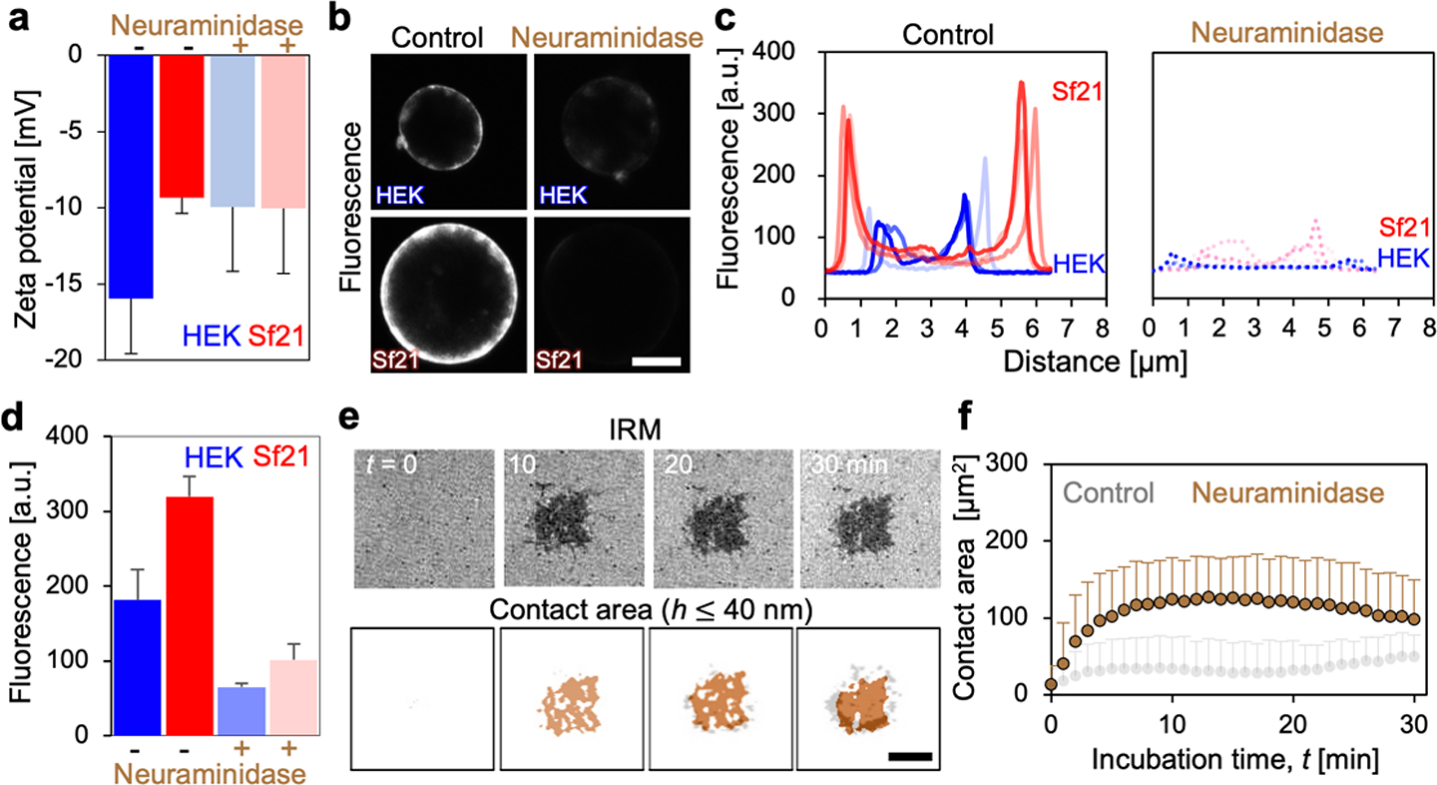
Surface potential of insect and mammalian cells and the molecular origins. (a) Zeta potential measurement of Sf21 and HEK cells after pretreatment with α2-3,6,8 neuraminidase (*n* = 5 cells). Error bars represent standard deviation. (b) Fluorescence confocal images of HEK and Sf21 cells after staining with fluorescent-WGA. (c) Fluorescence intensity profiles of Sf21 and HEK cells before and after treatment with α2-3,6,8 neuraminidase (*n* = 3 cells) and the (d) statistical analysis of peak fluorescence intensity. (e) IRM and contact area images of Sf21 after pretreatment with α2-3,6,8 neuraminidase. The contact area images of Sf21 in Figure 1b are shown again for better orientation (gray color). The scar bar corresponds to 10 μm. (f) Statistical analysis of the contact area (*h* ≤ 40 nm) of insect cells before and after treatment with α2-3,6,8 neuraminidase. The error bars represent the standard deviation (*n* = 5 cells). The gray plot (control) is a repeat of Figure 1c (red) for better orientation.

To characterize the spatial potential distribution of insect cell adhesion, positively and/or negatively charged substrates were prepared by silane coupling (see method details in the Supporting Information, Figure S3a). Previous studies have shown that a positively charged substrate (terminal group −NH_2_) leads to symmetry breaking of adhesion with a large spreading area,^25,26^ possibly via glycoconjugates with negative potential. Interestingly, the positively charged substrate did not increase the contact area of Sf21 cells and the presence of local patch-like structures at the cell periphery (∼1 μm) but suppressed ring-like structures (Figure 3a red; statistical analysis: Figure 3b red). In contrast, the negatively charged substrate significantly suppressed the adhesion and formation of ring-like patches (Figure 3a blue, statistical analysis: Figure 3b blue). These results clearly indicate that sugar structures with negative potential are homogeneously distributed on the cell surface, and the formation of local contacts at the cell periphery is driven by glycoconjugates with low electrostatic potential. To confirm the driving force for local segregation of glycoconjugates, we used 6-di-*o*-methyl-β- cyclodextrin (dMβCD) and methyl-β-cyclodextrin (MβCD) for the cells. Treatment with dMβCD and MβCD decreased the contact area of Sf21 cells due to the loss of segregation of glycoconjugates at the cellular periphery (Figure 3c; statistical analysis: Figure 3d). Previous reports have shown that dMβCD depletes phosphatidylethanolamine (PE) and cholesterol from the cellular membrane,^27^ but MβCD specifically depletes cholesterol from the cell surface.^28,29^ Moreover, gentle treatment of lovastatin (specific inhibitors for HMG-CR) also confirmed a decrease in the contact area and induced the disappearance of adhesion rings (Figure S3b,c). The coexistence of cholesterol and lipid leads to the formation of a lipid raft,^30,31^ such that the raft-like structure of glycoconjugates supports adhesion signatures at the cellular periphery. Interestingly, the small patch-like structures (∼1 μm) were also influenced by the depletion of cholesterol, but the positively charged substrate did not influence the presence at the cell periphery. The results indicate that small patch-like structures at the cell periphery are supported by the cholesterol with a negatively charged surface. To date, the raft structures of mammalian cells are generally invisible to conventional microscopy because their size is below the diffraction limit.^32^ Therefore, fluorescence lifetime imaging is commonly used to resolve small lipid raft structures at focal adhesion patches^33^ even though the spatial resolution is lower. To the best of our knowledge, the large local raft structure of the sugar moiety visible in this study’s experiment is the first to be visualized by conventional microscopy, except for phase separation with membrane models. These results clearly indicate that glycoconjugates with low surface potential at the cell periphery are supported by cholesterol and serve as a graft for a glycoconjugate. To elucidate the physical properties of the glycoconjugate raft, we examined membrane fluidity.^34^ The tight contact region (*h* ≤ 40 nm) has lower generalized polarization values (GP) (Figure 3e) than the weak contact region (*h* = 40–80), suggesting a “fluidic sugar raft” at the cell periphery. A previous report revealed that ordered domains of human monocyte cell lines were suppressed by the cholesterol depletion;^35^ however, the contribution of local domains to cell–substrate interfaces was not quantitatively assessed. In contrast, IRM accurately focuses on the cell–substrate interfaces and simultaneous imaging by Laurdan microscopy detected disordered tight contract areas. Treatment of MβCD suppressed the formation of adhesion ring with decreasing GP values (Figure S3d) indicating cholesterol support the formation of rings. A previous model membrane study detected higher membrane fluidity compared to the detached region with local curvature.^36^ Such a topological effect of cell membrane possibly induces fluidic peripheral adhesion rings that are tightly contacted to the substrate. Indeed, current knowledge of such glycolipid-enriched rafts is limited because of the inherent difficulties in their characterization. A limited number of studies have characterized glycolipid-enriched domains using grazing-incidence X-ray diffraction (GIXD), and phase behavior is determined by headgroup chemistry and by the length and saturation of the tails.^37^ Although further efforts need to be made to determine the lipid structures, lipid content, and physical properties of the cell membrane using various techniques^38,39^ and model systems to assess the fluidity of dynamical adhesion of giant unilamellar vesicle (GUV),^40^ a fingerprint of dynamic insect cell adhesion is supported by the raft of glycoconjugates.

**Figure 3:**
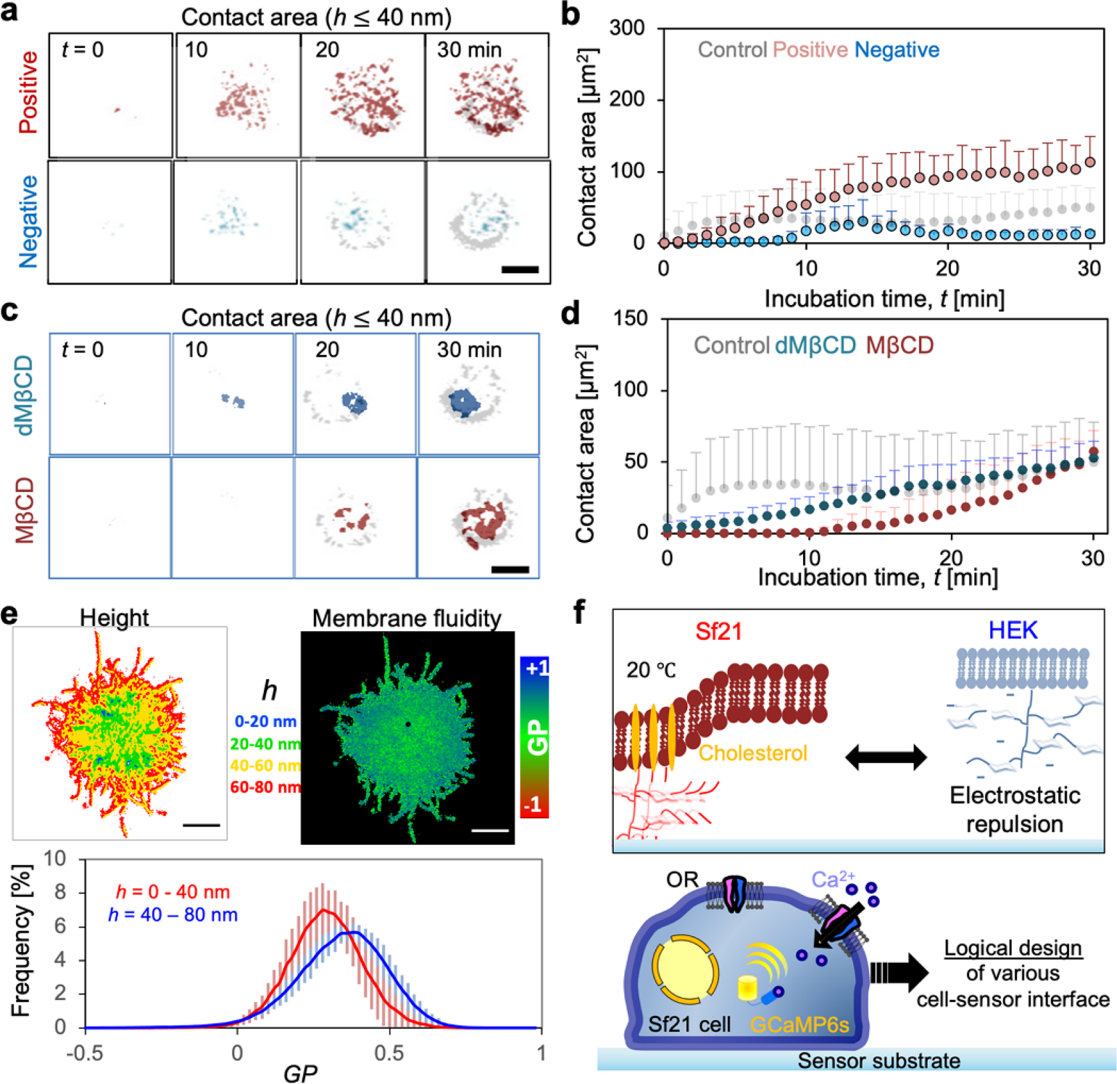
Effect of the electrostatic potential of substrate and cholesterol depletion on cell adhesion. Time-lapse images of the cropped contact area (*h* ≤ 40 nm) of Sf21 cells on (a) charged substrate and (b) corresponding statistical analysis of contact area. (c) Time-lapse images of the cropped contact area of Sf21 cells after pretreatment with dMβCD/MβCD and (d) corresponding statistical analysis of the contact area (*n* = 5 cells). The gray plot (control) is a repeat of Figure 1c (red) for better orientation. (e) Reconstructed height profiles and membrane fluidity of the cell membrane. The right panels show the histogram of GP values with different height thresholds (*n* = 10 cells). The results indicate that the region of close contact (0 ≤ *h* nm ≤40) is a lowly ordered membrane with high fluidity. (f) Schematic representation of the difference between the surface of insect and mammalian cells. The lower panels show schematic representations of the core sensory element within the FET odorant sensor and future research expansion. The scar bar corresponds to 10 μm.

In conclusion, cells adhered more strongly to the substrate immediately after the cells were placed on the substrate than HEK cells at ambient temperature. Zeta potential measurements identified a weak surface potential for Sf21 cells compared to HEK cells, which was not affected by the enzymatic digestion of surface sialic blushes. Fluorescent staining of *N*-acetyl glucosamine and sialic acid by WGA clearly showed a higher amount of Sf21 glycoconjugates than in HEK cells, but the electrostatic charge was weaker. To elucidate the molecular mechanism of ring-like glycoconjugate formation at the cell periphery with small patch-like structures, cholesterol depletion and inhibition of cholesterol synthesis (Figure S3b,c) were used to significantly suppress the adhesion signatures. Besides, membrane fluidity measurement identified highly fluidic signatures of the tight contact region compared to the detached region (Figure 3e and Figure S3d) which may be supported by membrane topology. These results suggest a new molecular structure of the insect cell interface; the formation of a glycoconjugate raft with low electrical potential leads to stronger adhesion (Figure 3c). Sf21cells showed the lowest electric potential and strong adhesion strength compared to other insect cell lines (Figure S3e). Therefore, using a genetically modified organism (Sf21 cells) with a stable and strong adhesion potential in “insect” cell-coupled FET devices is more advantageous over than the ones of mammals. In addition, our experimental platform can broaden the scope of visualizing the “living” cell-sensor interface quantitatively. Simultaneous reflection and fluorescence imaging provide accurate information for cell-adhesion interface by responding to a pheromone of silkmoth (bombykal) (Figure S3f) Visualization of cell adhesion interfaces are key for amplification of efficiency of electrical signal transfer,^5,6^ thus we believe that the electrical theory that accounts for real adhesion interfaces opens the possibility of rational design of cell-coupled FET biosensors.

## Supporting Information Available

Detailed experimental methods, optical setup, image analysis procedures, and figures for supporting each main figure (PDF)
Transparent Peer Review report available (PDF)
