# Supplementary material for: Low Surface Potential with Glycoconjugates Determines Insect Cell Adhesion at Room Temperature
Source: J Phys Chem Lett. 2022 Oct 6;13(40):9494–500. doi: 10.1021/acs.jpclett.2c01673 (PMC9575668; doi:10.1021/acs.jpclett.2c01673)
Supplement: Supplementary file 1 — jz2c01673_si_001.pdf [file jz2c01673_si_001.pdf]

Supporting information, SI

Title

Low Surface Potential with Glycoconjugates Determines Insect Cell Adhesion at Room-Temperature.

Authors

Takahisa Matsuzaki[1-3*], Daigo Terutsuki[4,5*], Shoma Sato[6], Kohei Ikarashi[6], Kohei Sato[7-10], Hidefumi Mitsuno[4], Ryu Okumura[11-13], Yudai Yoshimura[1], Shigeyoshi Usami[14], Yusuke Mori[14], Mai Fujii[6], Shota Takemi[15], Seiichiro Nakabayashi[3,6], Hiroshi Y. Yoshikawa,[1] and Ryohei Kanzaki[4]

Affiliations

[1]Center for Future Innovation, Graduate school of engineering, Osaka University, 2-1, Yamadaoka, Suita, Osaka 565-0871, Japan.

[2]Department of Applied Physics, Graduate School of Engineering, Osaka University, Suita, Osaka 565-0871, Japan.

[3]Division of Strategic Research and Development, Saitama University, Shimo-Okubo 255, Sakura-Ku, Saitama 338-8570, Japan.

[4]Research Center for Advanced Science and Technology, The University of Tokyo, 4-6-1 Komaba, Meguro-Ku, Tokyo 153-8904, Japan.

[5]Department of Finemechanics, Graduate School of Engineering, Tohoku University, 6-6-01 Aramaki-aza Aoba, Aoba-Ku, Sendai, 980-8579 Japan.

[6]Department of Chemistry, Saitama University, Shimo-Okubo 255, Sakura-ku, Saitama 338-8570, Japan.

[7]Graduate School of Science and Technology, Shizuoka University, 3-5-1 Johoku, Hamamatsu, Shizuoka, Japan.

[8]Course of Applied Chemistry and Biochemical Engineering, Department of Engineering, Graduate School of Integrated Science and Technology, Shizuoka University, 3-5-1 Johoku, Hamamatsu, Shizuoka, Japan

[9]Department of Applied Chemistry and Biochemical Engineering, Faculty of Engineering, Shizuoka University, Shizuoka, Japan.

[10]Research Institute of Green Science and Technology, Shizuoka University, 3-5-1 Johoku, Hamamatsu, Shizuoka, Japan

[11]Department of Microbiology and Immunology, Graduate School of Medicine, Osaka University, Osaka 565-0871, Japan.

[12]WPI Immunology Frontier Research Center, Osaka University, Osaka 565-0871, Japan.

[13]Integrated Frontier Research for Medical Science Division, Institute for Open and Transdisciplinary Research Initiatives, Osaka University, Osaka 565-0871, Japan.

[14]Division of Electrical, Electronic and Info communications Engineering, Graduate School of Engineering, Osaka University, Suita, Osaka 565-0871, Japan.

[15]Area of Regulatory Biology, Division of Life Science, Graduate School of Science and Engineering, Saitama University, 255 Shimo-Okubo, Sakura-Ku, Saitama 338-8570, Japan.

Contact Address

*Double first and corresponding authors.

Email: matsuzaki@ap.eng.osaka-u.ac.jp, Tel: +81-6-6879-7838

Email: daigo.terutsuki.a7@tohoku.ac.jp, Tel: +81-22-795-3586

Methods

Materials and reagents. Deionized water from a Milli-Q device (Japan Millipore Ltd., Tokyo, Japan) was used in this study. Unless otherwise stated, all other chemicals were purchased from Wako (Tokyo, Japan) or Life Technologies (Tokyo, Japan) and used without further preparation.

Cell Culture and chemical pretreatment. For the subculture of Sf21, the cells were cultured for 3 d in grace insect medium (Gibco, Tokyo, Japan) supplemented with 10 % fetal bovine serum (FBS), 1 % v/v penicillin-streptomycin, and 1 % v/v gentamicin. Sf21 cells were incubated in atmospheric CO_2 concentration at 27 °C. The cells were freshly prepared by manual pipetting (without any trypsin), and 3×10^5 cells were spread in 2 mL of culture medium on a 3.5 cm plastic dish (Thermo, Tokyo, Japan, 3×10^5 cells /9 cm^2). To spread the cells on the glass substrate, they were rinsed with 1 mL of PBS solution (Nissui, Tokyo, Japan) and centrifuged (200 × G, 3 min, 4 °C) to remove the protein content from the solution. Then, 200 µL of the resuspended PBS solution (3×10^5 cells) was spread onto a glass-bottom dish (Iwaki, Tokyo, Japan). For the subculture of HEK cells, the cells were cultured for 3 days in Dulbecco's modified Eagle's medium (DMEM) supplemented with 10% FBS, 1 % v/v penicillin-streptomycin, and 1 % *v/v* in 5 % CO_2 incubator at 37°C. It should be noted that both cell lines were subcultures with keeping native adherent state [1, 2]. After reaching confluence, the HEK293T cells were removed with trypsin. The 6-di-*o*-methyl-β- cyclodextrin (dMβCD) and methyl-β-cyclodextrin (MβCD) solutions were reconstituted from the powder, and a 10 mM RPMI 1640 solution was applied to the cells and allowed to react for 30 min at 20 °C.

Preparation of positively/negatively charged glass substrate by silane coupling reaction. We followed the established protocols for silane coupling[3]. The circular glass slips (Iwaki, Shizuoka, Japan) were washed according to the RCA cleaning method. Briefly, the glass slips were sonicated in ethanol, methanol, and MilliQ for 3 min. Mixtures of 4 v/v % NH_3 *aq* and 5 v/v % H_2O_2 were heated for 30 min at 60 °C. The solution was then baked on a heat plate, and the toluene solution was poured off. APTES (1 v/v % amino-propyl-triechoxysilane (APTES)) was reacted at room temperature for 30 min to form a positively charged surface (**Figure S3a**). The addition of succinic anhydride solution to amino-terminated glass results in a negatively charged surface.

Visualization of the cell-substrate interface by IRM and analysis. Unless stated otherwise, IRM of the cells was obtained in a solution of PBS (Ca^{2+} free, 137 mM NaCl, 2.7 mM KCl, 8 mM Na_2HPO_4, 1.4 mM KH_2PO_4, Nissui, Tokyo, Japan). The IRM was used to visualize the cell-substrate interface. The IRM used objective lenses such as SR HP Plan Apoλ (silicon immersion oil, numerical aperture = 1.25, ×100, Nikon, Tokyo, Japan) or Plan Apoλ (numerical aperture = 1.25, ×40, Nikon, Tokyo, Japan). A continuous-wave laser was used as the light source for the confocal systems (C2-Nikon TiE or A1R-Nikon-Ti-E). To obtain IRM images, a 20/80 beam splitter was used in the photomultiplier (PMT) channels. An intensity threshold according to previous protocols[4, 5] was used to calculate the contact zone. Regions with pixel intensities, $I\chi$, satisfying the following equation within the cell contour were defined as the contact area.

$$I_\chi \leq \chi(I_{\max} - I_{\min}) + I_{\min} \quad (1)$$

where χ is the threshold factor (0−1). The maximum intensity I_{max} was carefully determined from the interference fringes at the cell periphery. The minimum intensity within the contour was considered I_{min}. To reconstruct the height profiles of cells, we used the following equations[6]:

$$\frac{2I - (I_{max} + I_{min})}{-(I_{max} + I_{min})} = \cos\left(\frac{4\pi n}{\lambda} h\right) \quad (2)$$

Here, I is the measured intensity and I_{max} and I_{min} are the maximum and minimum intensities, respectively. where λ is the wavelength of the illumination light (546 nm), h is the distance between

the cells and the substrate, and n is the refractive index of the medium (~1.333). Fresh glass bottom dishes (Iwaki, Shizuoka, Japan) were used, and the (~3 × 10^5 cells/dish) was seeded. The analysis described above was performed using Fiji software.

Measurement of Zeta potential. Cells (~3 × 10^5 cells/mL) were suspended in PBS solution, and the zeta potential was measured using a conventional zeta potential analyzer (ZetaPALS, Brookhaven Inst, New York, United States). The potential parameter was fitted using built-in software.

Sialidase reaction and sialic staining. According to a previously described protocol[7], sialidase enzymes were reacted with cells (~3 × 10^5 cells / 10 µL RPMI solution) in the presence of 600U α2-3,6,8 neuraminidase(New England Bio Lab Inc., Tokyo, Japan) for 15 min at 37 °C. The reacted cells were then poured onto a substrate. Sialic blushes were stained with wheat germ agglutinin (WGA, derived from *triticum vulgaris*, FITC conjugated, Vector Laboratories Inc., California, United States). WGA (1 µM) was added to the cells and incubated for 15 min at room temperature. The cells were washed with PBS, and the remaining fluorescent molecules were centrifuged to suppress background intensity.

Laurdan microscopy. To measure membrane fluidity, we followed a well-established protocol for staining and calculating membrane fluidity[8-11]. In brief, dimethyl-6-dodecanoyl-2-naphthylamine (Laurdan, AdipoGen Life Science, California, United States) was selected as the fluidity probe and dissolved in DMSO solution (9 mM final). The final concentration of the laurdan probe was constant (30 µ M) in RPMI 1640 medium. Cells were incubated in the media for 10-30 min at 20 °C and then observed with a confocal microscope (A1-Nikon-TiE or C2) equipped with a femtosecond laser light source (Chameleon, λ= 800 nm) or continuous wave laser (Coherent inc, 100 mW, λ= 408 nm). Fluorescence intensity at λ= 408–458 nm ($I_{408\text{-}458}$) or λ= 468–532 nm ($I_{468\text{-}532}$) was measured using a GaAsP or PMT detector inside the confocal unit. To evaluate cell membrane fluidity, GP values were defined as

$$GP = \frac{I_{408-458} - G \times I_{468-532}}{I_{408-458} + G \times I_{468-532}} \quad (3)$$

and G values are calibration factors and defined as:

$$G = \frac{GP_{meas} + 1}{GP_{meas} - 1} \div \frac{GP_{ref} + 1}{GP_{ref} - 1} \quad (4)$$

GP_{ref} = 0.207 was used as the reference GP for Laurdan. The measured GP of laurdan in DMSO solution (30 µM final concentration) was used for GP_{meas} using G = 1 in equation 3. The above-described analysis was performed with the Fiji software or self-built macro on the Igor PRO.

Supporting Figures

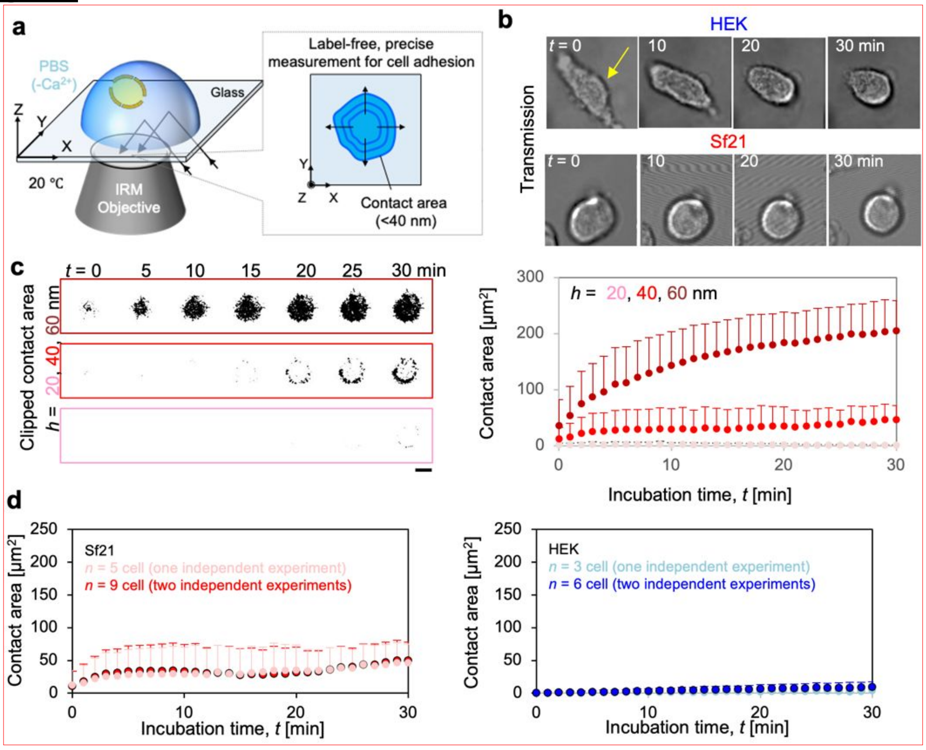

Figure S1: Detailed contact area analysis for Sf21 as a control. (Extended data for Figure 1). (a) Schematic of the experimental setup for evaluating the contact area of cells. (b) Representative transmission images of HEK and Sf21 cells. HEK cells show elongated morphology (yellow highlighted) arrow, but the actual contact area is smaller than that of Sf21. The results emphasize the importance of using IRM to visualize the cell adhesion to the substrate. (c) Truncated area of insect cells with different heights and corresponding statistical analysis (right panel, $n = 5$). The contact area ($h < 40$ nm) is optimal for detecting the adhesion signature of insect cells (*i.e.*, a narrow contact region around the cell periphery). The scar bar corresponds to 10 µm. (d) Two independent experiments to evaluate the contact area of Sf21 cell adhesion. Sf21 is established as a cell line, and the stability of adhesion signatures, morphological parameters, and fluorescence intensity responding to a pheromone of silkmoth was confirmed by our previous paper[12]. Both results support the high stability of our cell lines for the analysis of contact area.

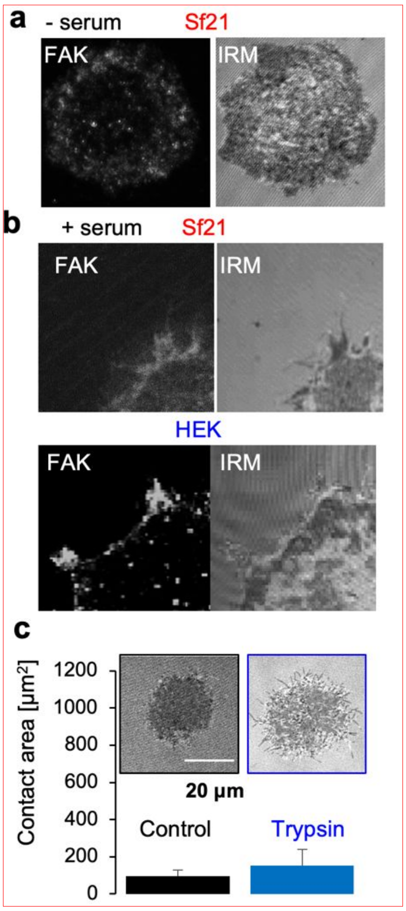

Figure S2: Influence of biological factors on insect cell adhesion and surface characterization of insect cells after treatment with neuraminidase (Extended data for Figure 2). (a) Fluorescence and reflectance images of Sf21 and HEK cells in the (a) absence and (b) presence of fetal bovine serum. Focal adhesion kinase (FAK) targeted antibody was from mammalians (Abcam, Heidelberg, Germany). (c) Contact area (h < 40 nm) analysis of Sf21 cells before and after treatment with trypsin. (n = 3).

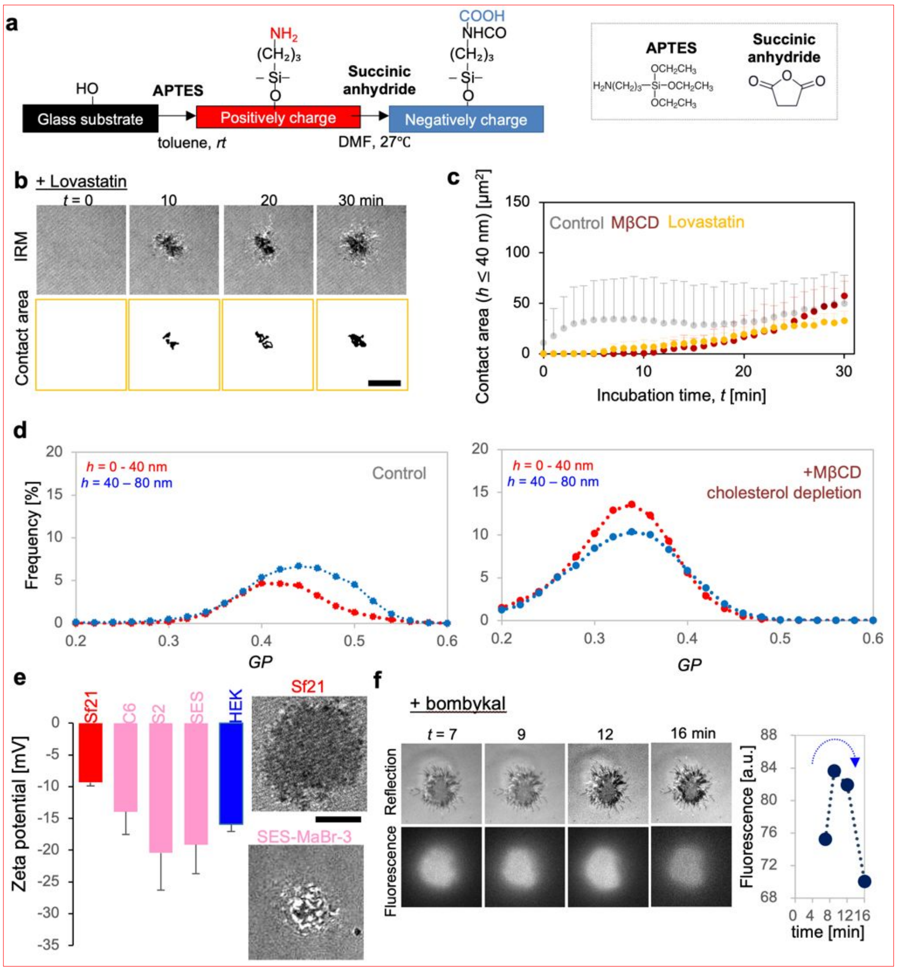

Figure S3: Quantitative evaluation of topological and membrane fluidity profiles of insect cell adhesion. (Extended data for Figure 3). (a) Preparation of glass substrates with different charge by silane coupling. (b) Time course of representative IRM images and cropped contact area ($h \leq 40$ nm) of Sf21 cells after treatment of 1.5 μM lovastatin (inhibitor for cholesterol synthesis) for 3 days. (c) Statistical analysis of the corresponding contact area during incubation ($n = 5$). The error bar represents the standard deviation. (d) Representative histograms of GP (Generalized polarization) before and after treatment of 10 mM MβCD. Region of tight contact region ($h = 0 – 40$ nm) and detached region ($h = 40 – 80$ nm) is displayed in red and blue color respectively. (e) Zeta potential analysis of different insect cell lines, C6/36: albopictus, S2: Drosophila, SES-MaBr-3: *Mamestra brassicae*. Representative reflectance images of SES-MaBr-3 are shown in the right panels. The scar bar corresponds to 10 μm. (f) Representative IRM and fluorescence images of Sf21 cells after adding 10 μM bombykal. Here, Sf21 cells expressing BmOR3, which specifically responds to bombykal, were used, and the fluorescence from GCaMP6 was monitored. Detailed protocol for the establishment of BmOR3 positive Sf21 cell lines was described in our previous papers[12].

Supporting references

(1) Malm, M.; Saghaleyni, R.; Lundqvist, M.; Giudici, M.; Chotteau, V.; Field, R.; Varley, P. G.; Hatton, D.; Grassi, L.; Svensson, T.; et al. Evolution from adherent to suspension: systems biology of HEK293 cell line development. *Sci. Rep.* **2020**, *10* (1), 18996.

(2) Vaughn, J.; Goodwin, R.; Tompkins, G.; McCawley, P. The establishment of two cell lines from the insectspodoptera frugiperda (lepidoptera; noctuidae). *In vitro* **1977**, *13* (4), 213-217.

(3) Syga, L.; Spakman, D.; Punter, C. M.; Poolman, B. Method for immobilization of living and synthetic cells for high-resolution imaging and single-particle tracking. *Sci. Rep.* **2018**, *8* (1), 13789.

(4) Matsuzaki, T.; Ito, K.; Masuda, K.; Kakinuma, E.; Sakamoto, R.; Iketaki, K.; Yamamoto, H.; Suganuma, M.; Kobayashi, N.; Nakabayashi, S.; et al. Quantitative Evaluation of Cancer Cell Adhesion to Self-Assembled Monolayer-Patterned Substrates by Reflection Interference Contrast Microscopy. *J. Phys. Chem. B* **2016**, *120* (7), 1221-1227.

(5) Matsuzaki, T.; Sazaki, G.; Suganuma, M.; Watanabe, T.; Yamazaki, T.; Tanaka, M.; Nakabayashi, S.; Yoshikawa, H. Y. High Contrast Visualization of Cell-Hydrogel Contact by Advanced Interferometric Optical Microscopy. *J. Phys. Chem. Lett.* **2014**, *5* (1), 253-257.

(6) Limozin, L.; Sengupta, K. Quantitative reflection interference contrast microscopy (RICM) in soft matter and cell adhesion. *ChemPhysChem* **2009**, *10* (16), 2752-2768.

(7) Xiao, H.; Woods, E. C.; Vukojicic, P.; Bertozzi, C. R. Precision glycocalyx editing as a strategy for cancer immunotherapy. *Proc. Natl. Acad. Sci. U S A* **2016**, *113* (37), 10304-10309.

(8) Gaus, K.; Gratton, E.; Kable, E. P.; Jones, A. S.; Gelissen, I.; Kritharides, L.; Jessup, W. Visualizing lipid structure and raft domains in living cells with two-photon microscopy. *Proc. Natl. Acad. Sci. U S A* **2003**, *100* (26), 15554-15559.

(9) Owen, D. M.; Rentero, C.; Magenau, A.; Abu-Siniyeh, A.; Gaus, K. Quantitative imaging of membrane lipid order in cells and organisms. *Nat. Protoc.* **2011**, *7* (1), 24-35.

(10) Parasassi, T.; De Stasio, G.; Ravagnan, G.; Rusch, R.; Gratton, E. Quantitation of lipid phases in phospholipid vesicles by the generalized polarization of Laurdan fluorescence. *Biophysical journal* **1991**, *60* (1), 179-189.

(11) Matsuzaki, T.; Matsumoto, S.; Kasai, T.; Yoshizawa, E.; Okamoto, S.; Yoshikawa, H. Y.; Taniguchi, H.; Takebe, T. Defining Lineage-Specific Membrane Fluidity Signatures that Regulate Adhesion Kinetics. *Stem Cell Reports* **2018**, *11* (4), 852-860.

(12) Terutsuki, D.; Mitsuno, H.; Sakurai, T.; Okamoto, Y.; Tixier-Mita, A.; Toshiyoshi, H.; Mita, Y.; Kanzaki, R. Increasing cell–device adherence using cultured insect cells for receptor-based biosensors. *Royal Society open science* **2018**, *5* (3), 172366.
