# Supplementary material for: Low Surface Potential with Glycoconjugates Determines Insect Cell Adhesion at Room Temperature
Source: J Phys Chem Lett. 2022 Oct 6;13(40):9494–500. doi: 10.1021/acs.jpclett.2c01673 (PMC9575668; doi:10.1021/acs.jpclett.2c01673)
Supplement: Supplementary file 2 — jz2c01673_si_002.pdf [file jz2c01673_si_002.pdf]

jz-2022-01673n.R1

Name: Peer Review Information for "Low Surface Potential with Glycoconjugates Determines Insect Cell Adhesion at Room-Temperature."

First Round of Reviewer Comments

Reviewer: 1

Comments to the Author

The group has already shown previously that exposure of the living insect cells expressing olfactory receptors to specific odorants can be sensed by the FET with high sensitivity and selectivity. In this work they show how the adhesion of the insect cell itself can be measured quantitatively using IRM. They make a comprehensive study of the adhesion pattern of insect cells (in comparison to mammalian cells). For the insect cells, they show the surface-charge dependence of the adhesion, the cholesterol-dependence and role of glycoconjugation in the adhesion pattern.

However, an important revision would be to address the rushed conclusion that the abolition of the adhesion-ring by cholesterol depletion indicates the that the ring is a micron-sized raft. Authors may consider the fact that acute depletion of cholesterol would have many downstream effect on membrane mechanics (increasing membrane tension see https://www.pnas.org/doi/abs/10.1073/pnas.2104820118) and signaling - thus affecting adhesion/interference pattern. Another clarification/explanation needed is why low GP values are measured at the raft-rich-regions (as assumed) while reports (https://www.pnas.org/doi/10.1073/pnas.2534386100) measure higher values. Could temperature be the reason? Authors may consider checking the effect of mbcd on GP values. Once validated the inference will indeed be new in the field. Authors must also consider the possibility of many small rafts being enriched at the periphery rather than a single big raft-patch.

The paper presents a very important advance in the combined use of IRM and FET sensors to address many challenging question. This work can also provide a deeper understanding about insect cell adhesion and its regulation by membrane glycosylation. However, for the complete impact of the work, it is essential that the authors also demonstrate sensing of odorants together with IRM - like in control, cholesterol depleted cells and after treatment with neuraminidase.

Some Technical suggestions:

a. Unit of area in fig 1 c is missing

b. Specify if n=3 : 3 cells or 3 independent repeats. At least three independent experimental repeats must be done

c. Fig 2 b must also be quantified. Mean peripheral intensity compared across cell populations with three repeats.

d. Quantification of effect of neuraminidase, mbcd and GP measurement must be accommodated in the main figures as part of existing figures

Author's Response to Peer Review Comments:

2, September 2022

Dear Professor Editor

We are sending our revised manuscript entitled "Low Surface Potential with Sugar Rafts Determine Insect Cell Adhesion at Room-Temperature (jz-2022-01673n) (thank you very much for extending the revision due to **Sept 2**). Reviewer 1 pointed out that "suppression of the adhesion ring by cholesterol depletion indicates the presence of raft-like structures" is rushed conclusions. For this revision, we carefully confirmed the suppression of adhesion rings with a specific inhibitor for cholesterol synthesis (**new Figure S3b-c**) and added the description of the possible origins of fluidic signatures (previous lines **174-182, new Figure S3d**). Besides, according to the reviewer's suggestion, we demonstrated simultaneous sensing odorants together with IRM (**new Figure S3f**). To encourage the reader's understanding, we included the "point of reviewer's concern" in the conclusion part (**blue color text**).

In conclusion, cells adhered more strongly to the substrate immediately after the cells were placed on the substrate than HEK cells at ambient temperature. Zeta potential measurements identified a weak surface potential for Sf21 cells compared to HEK cells, which was not affected by the enzymatic digestion of surface sialic blushes. Fluorescent staining of N-acetryl glucosamine and sialic acid by WGA clearly showed a higher amount of Sf21 glycoconjugates than in HEK cells, but the electrostatic charge was weaker. To elucidate the molecular mechanism of ring-like glycoconjugate formation with small patch-like structures *at the cell periphery, cholesterol depletion and inhibition of cholesterol synthesis (**Figure S3b-c**) were used to significantly suppress the adhesion signatures. Besides, membrane fluidity measurement identified highly fluidic signatures of the tight contact region compared to the detached region (**Figure 3e** and **Figure S3d**) which may be supported by membrane topology. These results suggest a new molecular structure of the insect cell interface; the formation of a glycoconjugate raft with low electrical potential leads to stronger adhesion (**Figure 3f**). Sf21cells showed the lowest electric potential and strong adhesion strength compared to other insect cell lines (**Figure S3e**). Therefore, using a genetically modified organism (Sf21 cell) with a stable and strong adhesion potential in "insect" cell-coupled FET devices is more advantageous over than the ones of mammals.* Simultaneous reflection and fluorescence imaging provide accurate information for cell-adhesion interface by responding to a pheromone of silkmoth (bombykal) (**Figure S3f**) *Visualization of cell adhesion interfaces are key for amplification of efficiency of electrical signal transfer[5-6], thus we believe that the electrical theory that accounts for real adhesion interfaces opens the possibility of rational design of cell-coupled FET biosensors.*

Although the additional experiments took longer time than we expected, we are now sure that the logical flows and conclusions of our paper are convincing. We also corrected typographical errors. For clarity, the changes are summarized in our point-by-point answers named "Response to reviewer.pdf". All our changes in the main text and supplementary information were recorded using the review mode of word software in the files "main-revised.docx" and "SI-revised.docx". We also added revised manuscript without track: "main-revised (without track), SI-revised (without track).

Thanks to your "Supplementary Journal Cover Invitation", we enclosed two candidate of **cover art and legends**.

On the next page of this letter, we also attached "the Editor's Comments to Author" for a reminder of the previous review result. We deeply appreciate your thorough review for publication in *Journal of Physical Chemistry Letters.* Thank you very much for your consideration in advance. I am looking forward to hearing from you.

・**Takahisa Matsuzaki**
Assistant Professor, Graduate School of Engineering, Department of Applied Physics
Osaka University
2-1, Yamadaoka, Suita, Osaka 565-0871, Japan.
Phone: +81-6-6879-7838 Mail: matsuzaki@ap.eng.osaka-u.ac.jp
・**Daigo Terutsuki**
Assistant Professor, Graduate School of Engineering, Department of Finemechanics
Tohoku University
6-6-01 Aramaki-aza Aoba, Aoba-ku, Sendai, 980-8579 Japan.
Phone: +81-22-795-3586 Mail: daigo.terutsuki.a7@tohoku.ac.jp

Reviewer: 1
Recommendation: This paper is probably publishable, but major revision is needed; I do not need to see future revisions.

Comments:
The group has already shown previously that exposure of the living insect cells expressing olfactory receptors to specific odorants can be sensed by the FET with high sensitivity and selectivity. In this work they show how the adhesion of the insect cell itself can be measured quantitatively using IRM. They make a comprehensive study of the adhesion pattern of insect cells (in comparison to mammalian cells). For the insect cells, they show the surface-charge dependence of the adhesion, the cholesterol-dependence and role of glycoconjugation in the adhesion pattern.

However, an important revision would be to address the rushed conclusion that the abolition of the adhesion-ring by cholesterol depletion indicates the that the ring is a micron-sized raft. (Q1-1) Authors may consider the fact that acute depletion of cholesterol would have many downstream effect on membrane mechanics (increasing membrane tension see https://www.pnas.org/doi/abs/10.1073/pnas.2104820118) and signaling - thus affecting adhesion/interference pattern. (Q1-2) Another clarification/explanation needed is why low GP values are measured at the raft-rich-regions (as assumed) while reports (https://www.pnas.org/doi/10.1073/pnas.2534386100) measure higher values. Could temperature be the reason? Authors may consider checking the effect of mbcd on GP values. Once validated the inference will indeed be new in the field. (Q1-3) Authors must also consider the possibility of many small rafts being enriched at the periphery rather than a single big raft-patch.

The paper presents a very important advance in the combined use of IRM and FET sensors to address many challenging question. This work can also provide a deeper understanding about insect cell adhesion and its regulation by membrane glycosylation. However, for the complete impact of the work, (Q2)it is essential that the authors also demonstrate sensing of odorants together with IRM - like in control, cholesterol depleted cells and after treatment with neuraminidase.

Some Technical suggestions:
a. Unit of area in fig 1 c is missing
b. Specify if n=3 : 3 cells or 3 independent repeats. At least three independent experimental repeats must be done
c. Fig 2 b must also be quantified. Mean peripheral intensity compared across cell populations with three repeats.
d. Quantification of effect of neuraminidase, mbcd and GP measurement must be accommodated in the main figures as part of existing figures

Additional Questions:
Urgency: High
Significance: High
Novelty: High
Scholarly Presentation: Moderate
Is the paper likely to interest a substantial number of physical chemists, not just specialists working in the authors' area of research?: Yes

Author replies for reviewer#1 (Summary):

We sincerely appreciate reviewer #1 for providing critical questions and insightful suggestions. According to the proposed question (labeled in red above), we prepared point-by-point responses below to satisfy a necessary revision (underlines).

We added data about the impact of lovastatin treatment (specific inhibitor for cholesterol synthesis, Q1-1, **new Figure S3b-c**), confirming that the formation of adhesion rings is suppressed via down-regulation of cholesterol synthesis. Besides, a previous report proposed a cholesterol-rich ordered domain (Q1-2, Reviewer 1 cited: Gaus,…, Jessup, *PNAS* 2003), but their optical focus was not set to the cell-substrate interface. We carefully described the first impact of using interference reflection microscopy (IRM) to set insect cell-substrate interface. We discussed the possible origins of disordered adhesion rings (previous lines 174-182, **new Figure S3d**). We also carefully considered small rafts around the large local raft and their physical characters (Q1-3, *e.g.,* previous lines 103-105, and 162-166). Moreover, we demonstrated sensing of odorants with IRM in the condition of control (Q2, **new Figure S3f**) with reformatting main and SI figures (some technical suggestions). We now believe these improvements assess the proposed revision and highlight important advances in combining IRM with FET sensors.

However, an important revision would be to address the rushed conclusion that the abolition of the adhesion-ring by cholesterol depletion indicates the that the ring is a micron-sized raft. (Q1-1)…Authors may consider the fact that acute depletion of cholesterol would have many downstream effect on membrane mechanics (increasing membrane tension see https://www.pnas.org/doi/abs/10.1073/pnas.2104820118) and signaling-thus affecting adhesion/interference pattern.

(A1-1) We tested an inhibitor for HMGCR (*i.e.,* Lovastatin) to suppress cholesterol synthesis, which is a much milder and specific condition than the use of cyclodextrin (Reviewer 1 cited: Cox,…, Martinac, *PNAS* 2021). Representative images of IRM show the disappearance of adhesion rings (Figure S3b) and the suppression of contact area (Figure S3c), a comparable effect of cyclodextrin use. We added the description in the main text (Previous Line 160-166). Thus, we confirmed the critical conclusion that adhesion signatures at the cell periphery are supported by cholesterol.

[Before] previous line 160-166

Treatment with dMβCD and MβCD decreased the contact area of Sf21 cells due to the loss of segregation of glycoconjugates at the cellular periphery (**Figure 3b red,** statistical analysis: **Figure S3c**). Previous reports have shown that dMβCD depletes phosphatidylethanolamine (PE) and cholesterol from the cellular membrane[27], but MβCD specifically depletes cholesterol from the cell surface[28-29]. The coexistence of cholesterol and lipid lead to the formation of lipid raft[30-31], such that adhesion signatures at the cellular periphery are supported by the raft-like structure of glycoconjugates.

[After]

Treatment with dMβCD and MβCD decreased the contact area of Sf21 cells due to the loss of segregation of glycoconjugates at the cellular periphery (**Figure 3c-d red**). Previous reports have shown that dMβCD depletes phosphatidylethanolamine (PE) and cholesterol from the cellular membrane[27], but MβCD specifically depletes cholesterol from the cell surface[28-29]. Moreover, gentle treatment of lovastatin (specific inhibitors for HMG-CR) also confirmed a decrease in the contact area and induced the disappearance of adhesion rings (**Figure S3b-c**). The coexistence of cholesterol and lipid leads to the formation of lipid raft[30-31], such that the raft-like structure of glycoconjugates supports adhesion signatures at the cellular periphery.

(Q1-2) Another clarification/explanation needed is why low GP values are measured at the raft-rich-regions (as assumed) while reports (https://www.pnas.org/doi/10.1073/pnas.2534386100) measure higher values. Could temperature be the reason? Authors may consider checking the effect of mbcd on GP values. Once validated the inference will indeed be new in the field.

(A1-2) Gaus et al. indicated that cholesterol depletion decreased GP values and suppressed the formation of the "ordered" domain (high GP) (Reviewer 1 cited: Gaus et al., *PNAS* 2003). However, their focus was not set on the cell adhesion interface. Our paper is the first report to finely visualize a tight contact area by interference reflection microscopy (IRM) and identify its disordered characters (low GP) at the "insect cell" -substrate interface. We also confirmed that cholesterol depletion decreased GP values of insect cells (**new Figure S3d**). Thus, cholesterol should support the ordering of raft structure. We think the tight contact region's higher fluidity than the weak contact region is attributed to the topological effect. Previous model membrane studies revealed that membrane fluidity of tightly contact areas is higher than that of detached regions with local curvature (Woodward et al., *BBA Biomembrane* 2018). Thus, the obtained low GP at the cell periphery was possibly attributed to the topological effect of the cell membrane. In the following paper, we will confirm the fluidity of tightly contact membranes by visualizing dynamical contact of GUV (Giant Unilamellar Vesicles, seen like in Goennenwein,.., Sackmann *Biophysical J.* 2003). We highlighted the impact of combining IRM with Laurdan microscopy and described the possible origins of tight contact areas with low GP in the main text (**Previous Line 174-182**).

[Before] previous line 174-182

The tight contact region (h < 40 nm) has lower generalized polarization values (*GP*) (**Figure S3d**) than the weak contact region (40 < h nm < 80), suggesting a "fluidic sugar raft" at the cell periphery. Indeed, current knowledge of such glycolipid-enriched rafts is limited because of the inherent difficulties in their characterization. A limited number of studies have characterized glycolipid-enriched domains using grazing-incidence X-ray diffraction (GIXD), and phase behavior is determined by headgroup chemistry and by the length and saturation of the tails[35]. Although further efforts need to be made to determine the lipid structures, lipid content, and physical properties of the cell membrane using various techniques [36], a fingerprint of dynamic insect cell adhesion is supported by the raft of glycoconjugates.

[After]

The tight contact region ($h \leq 40$ nm) has lower generalized polarization values (*GP*) (**Figure 3e**) than the weak contact region ($40 \leq h$ nm ≤ 80), suggesting a "fluidic sugar raft" at the cell periphery. The previous report revealed that ordered domains of human monocyte cell lines were suppressed by cholesterol depletion[35] However, the contribution of local domains to cell-substrate interfaces was not quantitatively assessed. In contrast, IRM accurately focuses the cell-substrate interfaces, and simultaneous imaging by Laurdan microscopy detected disordered tight contract areas. Treatment of MβCD suppressed the formation of adhesion ring with decreasing GP values (**Figure S3d**), indicating cholesterol support the formation of rings. A previous study using model membranes detected higher membrane fluidity compared to the detached region with local curvature [36]. Such topological effect of cell membrane possibly induces fluidic peripheral adhesion rings, which is tightly in contact to the substrate. Indeed, current knowledge of such glycolipid-enriched rafts is limited because of the inherent difficulties in their characterization. A limited number of studies have characterized glycolipid-enriched domains using grazing-incidence X-ray diffraction (GIXD), and phase behavior is determined by headgroup chemistry and the length and saturation of the tails[37]. Although further efforts need to be made to determine the lipid structures, lipid content, and physical properties of the cell membrane using various techniques [38] and model systems to assess fluidity of dynamical adhesion of giant unilamellar vesicle (GUV)[39], a fingerprint of dynamic insect cell adhesion is supported by the raft of glycoconjugates.

(Q1-3) Authors must also consider the possibility of many small rafts being enriched at the periphery rather than a single big raft-patch.

(A3) Thank you very much for your interesting suggestion. Interestingly, the minor patch-like rafts at the cell periphery (~1 μm) were suppressed by the "negatively" charged substrate and depletion of cholesterol (Figure 3a blue, c). In contrast, a "positively charged substrate keeps patch-like structures (Figure 3a red). These results indicate that the cholesterol supports small patch-like configurations, and the surface is slightly negatively charged. We highlight these interesting molecular insights in the manuscript (previous lines 103-105, 152-154,162-166 below).

[Before] previous line 103-105

Here, by changing the intensity threshold factor, the contact area (*h* < 40 nm) was optimized for evaluation of the peripheral strong adhesion zone (**Figure S1c**).

[After]

Here, by changing the intensity threshold factor, the contact area ($h \leq 40$ nm) was optimized for evaluation of the peripheral strong adhesion zone and small patch-like structures (~1 μm) (**Figure S1c**).

[Before] previous line 152-154

Interestingly, the positively charged substrate did not increase the contact area of Sf21 cells but suppressed ring-like structures (**Figure 3a red,** Statistical analysis: **Figure S3b**).

[After]

Interestingly, the positively charged substrate did not increase the contact area of Sf21 cells and the presence of local patch-like structures at the cell periphery (~1 μm) but suppressed ring-like structures (**Figure 3a red,** Statistical analysis: **Figure S3b**).

[Before] previous line 162-166

Previous reports have shown that dMβCD depletes phosphatidylethanolamine (PE) and cholesterol from the cellular membrane[27], but MβCD specifically depletes cholesterol from the cell surface[28-29]. The coexistence of cholesterol and lipid leads to the formation of lipid raft[30-31], such that adhesion signatures at the cellular periphery are supported by the raft-like structure of glycoconjugates.

[After]

Previous reports have shown that dMβCD depletes phosphatidylethanolamine (PE) and cholesterol from the cellular membrane[27], but MβCD specifically depletes cholesterol from the cell surface[28-29]. The coexistence of cholesterol and lipid leads to the formation of lipid raft[30-31], such that the raft-like structure of glycoconjugates supports adhesion signatures at the cellular periphery. Interestingly, the small patch-like structures (~1 μm) was also influenced by the depletion of cholesterol, but the positively charged substrate did not affect the presence at the cell periphery. The results indicate that the cholesterol supports small patch like structures at the cell periphery with a negatively charged surface.

(Q2)it is essential that the authors also demonstrate sensing of odorants together with IRM - like in control, cholesterol-depleted cells, and after treatment with neuraminidase.

(A2) Thank you very much for your critical suggestion. We added the new data about simultaneous IRM and fluorescence imaging in control conditions (**new Figure S3f**). We prepared Sf21 cell lines expressing BmOR3 (a sex pheromone receptor of silkmoth *Bombyx mori*, which response explicitly to bombykal). This cell line co-expresses the GCaMP6 inside cells. Thus, the addition of bombykal enhances the green fluorescence intensities of the cells. After adding bombykal (t = 0 min), fluorescence intensity was significantly increased and reached the maximum at t = 9 min (**new Figure S3f right**). We highlighted this point in the conclusion part (previous lines 193-199). These data indicate that IRM is a powerful tool to assess the exact cell-adhesion interface during odorant sensing and provide a deeper insight into electrical theory that accounts for natural adhesion interfaces opening the possibility of rational design of cell-coupled FET biosensors.

[Before] previous line 193-199

Therefore, using a genetically modified organism (Sf21) with a stable and strong adhesion potential in “insect” cell-coupled FET devices is more advantageous over than the ones of mammals. In addition, our experimental platform can broaden the scope of visualizing the “living” cell-sensor interface quantitatively. Visualization of cell adhesion interfaces is key for amplification of efficiency of electrical signal transfer[5-6], thus we believe that the electrical theory that accounts for real adhesion interfaces opens the possibility of rational design of cell-coupled FET biosensors.

[After]

Therefore, using a genetically modified organism (Sf21 cell) with a stable and strong adhesion potential in “insect” cell-coupled FET devices is more advantageous over than the ones of mammals. In addition, our experimental platform can broaden the scope of visualizing the “living” cell-sensor interface quantitatively. Moreover, simultaneous reflection and fluorescence imaging provides accurate information for cell-adhesion interface with responding to a pheromone of silkmoth (bombykal) (**Figure S3f**). Visualization of cell adhesion interfaces is key for amplification of efficiency of electrical signal transfer[5-6], thus we believe that the electrical theory that accounts for real adhesion interfaces opens the possibility of rational design of cell-coupled FET biosensors.

Some Technical suggestions:

a. Unit of area in fig 1 c is missing

b. Specify if n=3 : 3 cells or 3 independent repeats. At least three independent experimental repeats must be done

c. Fig 2 b must also be quantified. Mean peripheral intensity compared across cell populations with three repeats.

d. Quantification of effect of neuraminidase, mbcd and GP measurement must be accommodated in the main figures as part of existing figures

(a) We confirmed to correct typographical errors in the main manuscript and SI (*e.g.,* we added the unit in Figure 1c).

(b) We compared the deviation of contact area between two independent experiments for Sf21 and HEK293T cells (**new Figure S1d**). We confirmed the shape of the increase in contact area was comparable because our insect cell (Sf21) is not *in vivo* sample but a “cell line”. Besides, our previous paper also described that the standard deviation of fluorescence intensity changes after the addition of bombykal was also slight even in three independent experiments (Terutsuki et al., *R. Soc. Open Sci.* 2018.). We believe that both results highlight the stability of the cells, and three independent repeats is not necessary for this paper. To mild the reviewer’s concern, we added data in old Figure 1c, prepared new **Figure 1c** and specified the number of analyzed cells in the caption (*n* =6 HEK cells, *n* =9 Sf21 cells with two independent experiments). We also carefully described the stability of cells in the caption of the **new Figure S1d**.

(c) We quantified averaged peak fluorescence intensity at the cell membrane in the **new Figure 2d**. We moved the new Figure 2c from the SI for the reader's support.

(d) We accommodate the corresponding SI figures to revised main figures (**Figures 1-3**).

We displayed revised main, SI, and TOC figures (with captions) below to support the reviewer’s understanding. Thank you again for reviewer1 carefully checking the manuscript and SI materials.

Revised Main Figures

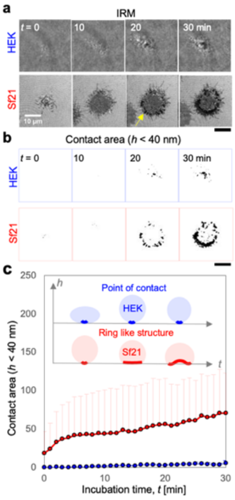
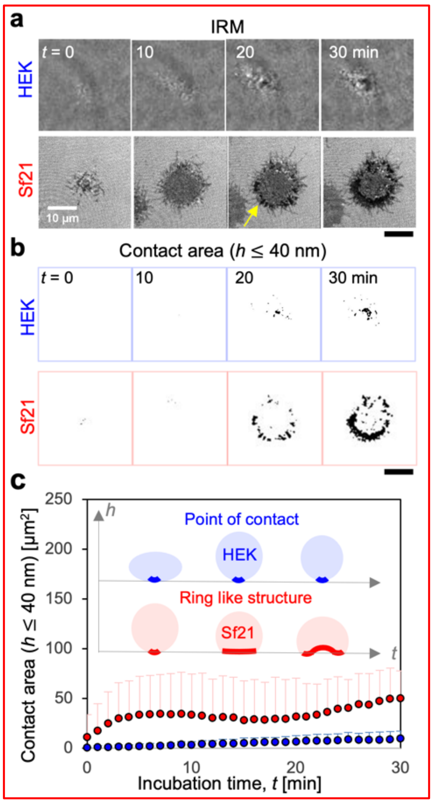

Before (left) and After (right) **Figure 1: Dynamic adhesion of insect and mammalian cells at room-temperature (20 °C).** Time course of (a) IRM images and (b) cropped contact area ($h \leq 40$ nm) of HEK and Sf21 cells. (c) Statistical analysis of the corresponding contact area during incubation (HEK: n = 6 cells, Sf21: n = 9 cells, two independent experiments for the analysis). The error bar represents the standard deviation. Inset shows a schematic representation of the adhesion morphology of Sf21 and HEK cells along the optical axis. The scar bar corresponds to 10 µm.

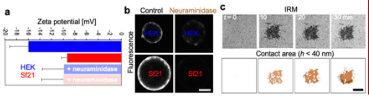
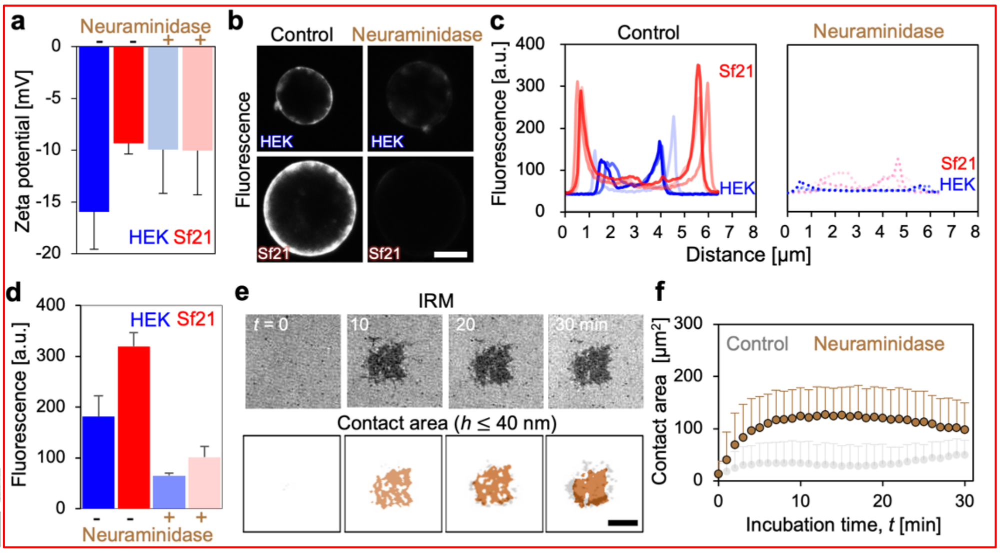

Before (left) of **Figure 2: The surface potential of insect and mammalian cells, and the molecular origins.** (a) Zeta potential measurement of Sf21 and HEK cells after pretreatment with α2-3,6,8 neuraminidase (n = 5). Error bars represent standard deviation. (b) Fluorescence confocal images of HEK and Sf21 cells after staining with fluorescent-WGA. (c) IRM and contact area images of Sf21 after pretreatment with α2-3,6,8 neuraminidase. The contact area images of Sf21 in Figure 1b are shown again for better orientation (gray color). The scar bar corresponds to 10 µm.

After (right) of **Figure 2: The surface potential of insect and mammalian cells, and the molecular origins.** (a) Zeta potential measurement of Sf21 and HEK cells after pretreatment with α2-3,6,8 neuraminidase (n = 5). Error bars represent standard deviation. (b) Fluorescence confocal images of HEK and Sf21 cells after staining with fluorescent-WGA. (d) Fluorescence intensity profiles of Sf21 and HEK cells before and after treatment with α2-3,6,8 neuraminidase (n = 3) and the (d) statistical analysis of peak fluorescence intensity. (e) IRM and contact

area images of Sf21 after pretreatment with α2-3,6,8 neuraminidase. The contact area images of Sf21 in Figure 1b are shown again for better orientation (gray color). The scar bar corresponds to 10 µm. (f) Statistical analysis of the contact area ($h \leq 40$ nm) of insect cells before and after treatment with α2-3,6,8 neuraminidase. The error bars represent the standard deviation ($n = 5$). The gray plot (control) is a repeat of **Figure 1c** (red) for better orientation.

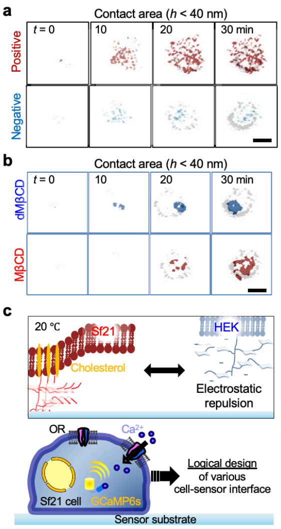

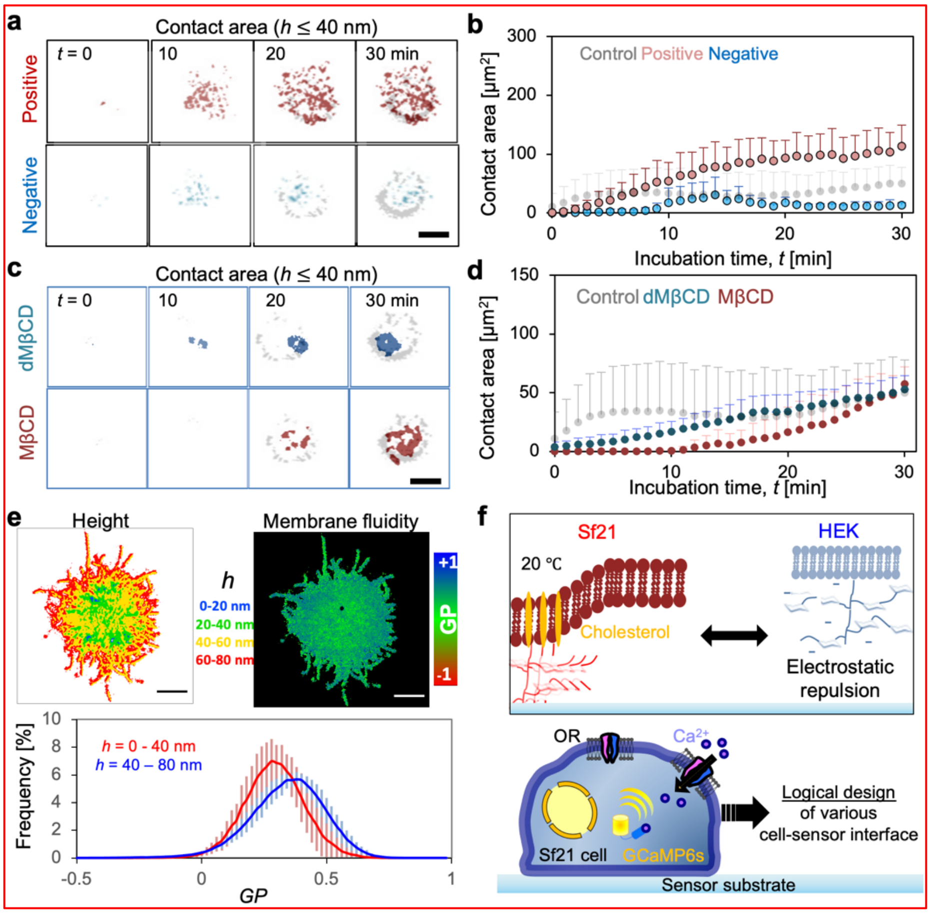

Before (left) of **Figure 3: Effect of electrostatic potential of substrate and cholesterol depletion on cell adhesion.** Time lapse images of the cropped contact area ($h < 40$ nm) of Sf21 cells on (a) charged substrate and (b) after pretreatment with dMβCD and MβCD. The contact area images of Sf21 in **Figure 1b** are shown again for better orientation (gray color). (c) Schematic representation of the difference between the surface of insect and mammalian cells. The lower panels show schematic representations of the core sensory element within the FET odorant sensor and future research expansion. The scar bar corresponds to 10 µm.

After (right) of **Figure 3: Effect of electrostatic potential of substrate and cholesterol depletion on cell adhesion.** Time lapse images of the cropped contact area ($h \leq 40$ nm) of Sf21 cells on (a) charged substrate and (b) corresponding statistical analysis of contact area. (c) Time lapse images of the cropped contact area of Sf21 cells after pretreatment with dMβCD and MβCD and (d) corresponding statistical analysis of the contact area ($n = 5$). The gray plot (control) is re-a repeat of **Figure 1c** (red) for better orientation. (e) Reconstructed height profiles and membrane fluidity of cell membra. The right panels show the histogram of GP values with different height thresholds ($n = 10$). The results indicate that the region of close contact ($0 \leq h$ nm≤ 40) is a low ordered membrane with high fluidity. (f) Schematic representation of the difference between the surface of insect and mammalian cells. The lower panels show schematic representations of the core sensory element within the FET odorant sensor and future research expansion. The scar bar corresponds to 10 µm.

Revised SI Figures

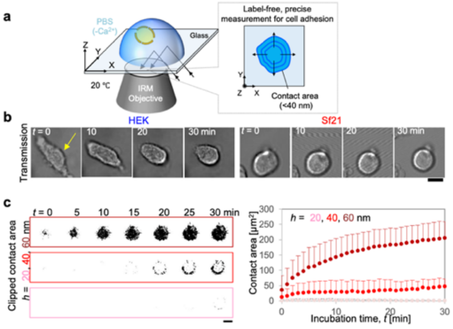

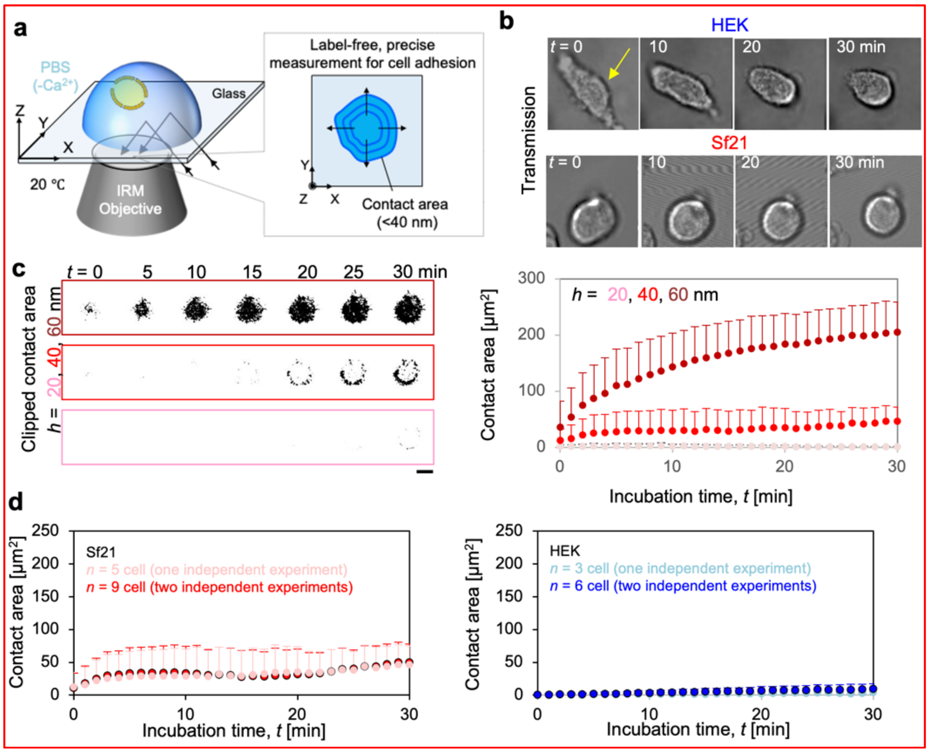

Before (left) of **Figure S1: Detailed contact area analysis for Sf21 as a control. (Extended data for Figure 1).** (a) Schematic of experimental setup for evaluating contact area of cells. (b) Representative transmission images of HEK and Sf21 cells. HEK cells show elongated morphology (yellow highlighted) arrow, but the actual contact area is smaller than that of Sf21. The results highlight the importance of using IRM to visualize the cell adhesion to the substrate. (c) Truncated area of insect cells with different heights and corresponding statistical analysis (right panel, $n = 5$). The contact area ($h < 40$ nm) is optimal for detecting the adhesion signature of insect cells (*i.e.*, a narrow contact region around the cell periphery). The scar bar corresponds to 10 μm.

After (left) of **Figure S1: Detailed contact area analysis for Sf21 as a control. (Extended data for Figure 1).** (a) Schematic of experimental setup for evaluating contact area of cells. (b) Representative transmission images of HEK and Sf21 cells. HEK cells show elongated morphology (yellow highlighted) arrow, but the actual contact area is smaller than that of Sf21. The results highlight the importance of using IRM to visualize the cell adhesion to the substrate. (c) Truncated area of insect cells with different heights and corresponding statistical analysis (right panel, $n = 5$). The contact area ($h <\leq$ 40 nm) is optimal for detecting the adhesion signature of insect cells (*i.e.*, a narrow contact region around the cell periphery). The scar bar corresponds to 10 μm. (d) Two independent experiments to evaluate the deviation of Sf21 and HEK contact area. Sf21 is established as a cell line and the stability of adhesion signatures, morphological parameters, and fluorescence intensity responding to a pheromone of silkmoth was confirmed by our previous paper[12]. Both results support the high stability of our cell lines for the analysis of contact area.

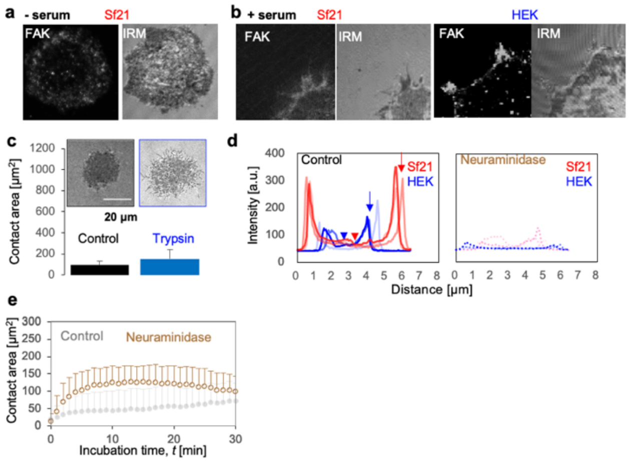

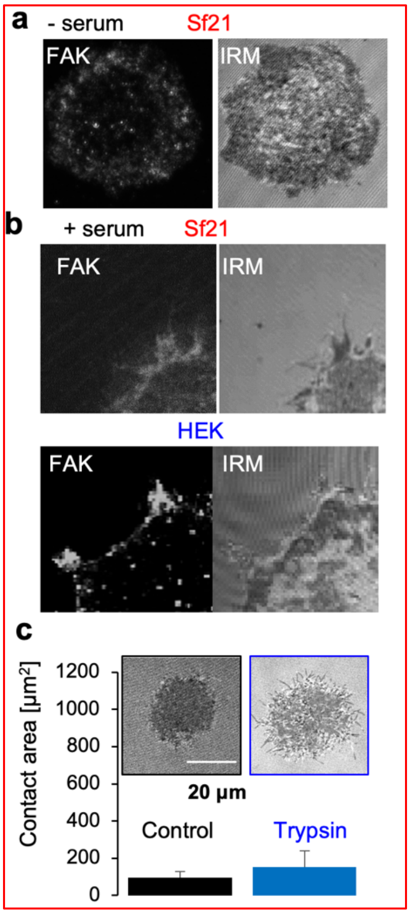

Before (left) of **Figure S2: Influence of biological factors on insect cell adhesion, and surface characterization of insect cells after treatment with neuraminidase (Extended data for Figure 2).** (a) Florescence and reflectance images of Sf21 and HEK cells in the (a) absence and (b) presence of fetal bovine serum. Focal adhesion kinase (FAK) targeted antibody was from mammalians (Abcam, Heidelberg, Germany). (c) Contact area (*h* < 40 nm) analysis of Sf21 cells before and after treatment with trypsin. (*n* = 3). (d) Fluorescence intensity profiles of Sf21 and HEK cells before and after treatment with α2-3,6,8 neuraminidase (raw fluorescence images; **Figure 2b**) (*n* = 3). The colored arrowhead and arrow indicate the center and cell membrane, respectively. (e) Statistical analysis of the contact area (*h* < 40 nm) of insect cells before and after treatment with α2-3,6,8 neuraminidase (raw fluorescence images; **Figure 2c**). The error bars represent the standard deviation (*n* = 5). The gray plot (control) is a repeat of **Figure 1c** (red) for better orientation.

After (right) of **Figure S2: Influence of biological factors on insect cell adhesion, and surface characterization of insect cells after treatment with neuraminidase (Extended data for Figure 2).** (a) Florescence and reflectance images of Sf21 and HEK cells in the (a) absence and (b) presence of fetal bovine serum. Focal adhesion kinase (FAK) targeted antibody was from mammalians (Abcam, Heidelberg, Germany). (c) Contact area (*h* < 40 nm) analysis of Sf21 cells before and after treatment with trypsin. (*n* = 3). ~~(d) Fluorescence intensity profiles of Sf21 and HEK cells before and after treatment with α2-3,6,8 neuraminidase (raw fluorescence images; **Figure 2b**) (*n* = 3). The colored arrowhead and arrow indicate the center and cell membrane, respectively. (e) Statistical analysis of the contact area (*h* < 40 nm) of insect cells before and after treatment with α2-3,6,8 neuraminidase (raw fluorescence images; **Figure 2c**). The error bars represent the standard deviation (*n* = 5). The gray plot (control) is a repeat of **Figure 1c** (red) for better orientation.~~

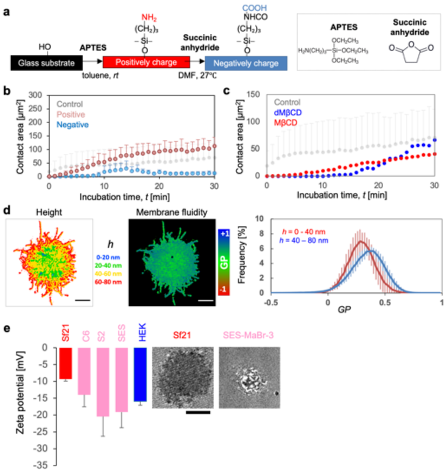

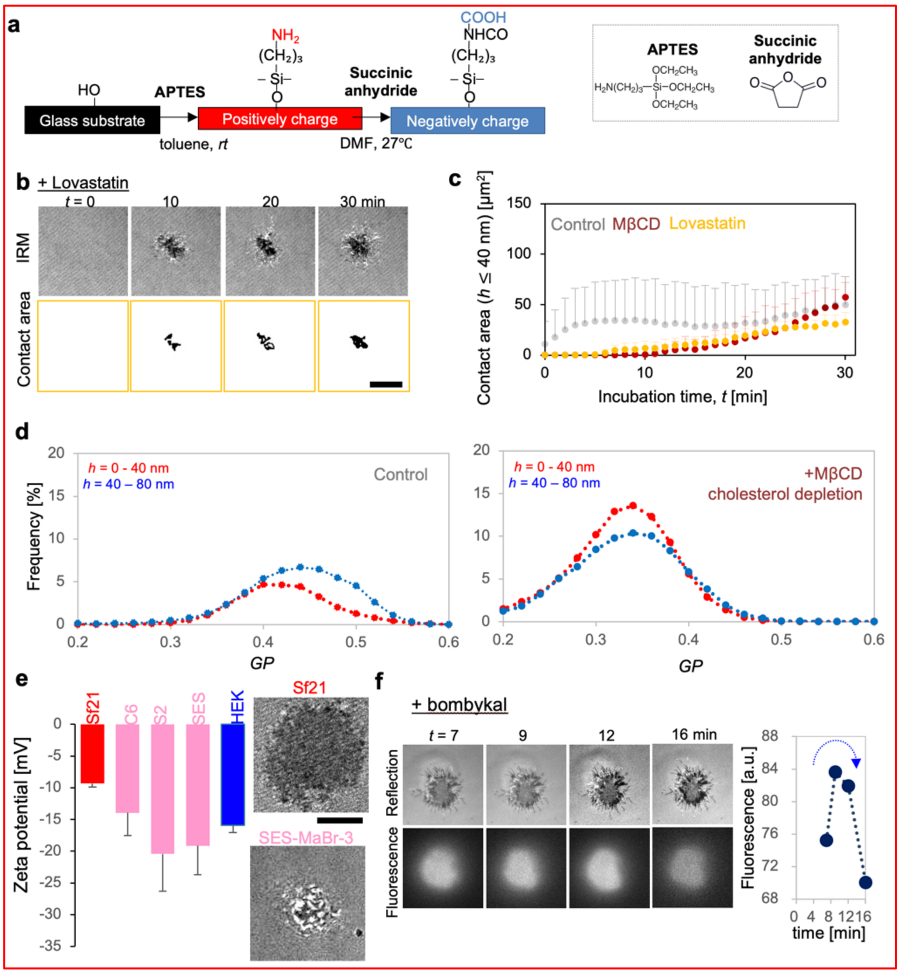

Before (left) Figure S3: Quantitative evaluation of topological and membrane fluidity profiles of insect cell adhesion. (Extended data for Figure 3). (a) Preparation of glass substrates with different charges by silane coupling. Statistical analysis of the contact area (h < 40 nm) of Sf21 cells (b) on the charged substrate and (c) pretreatment with dMβCD and MβCD (n = 5). The gray plot (control) is a repeat of **Figure 1c** (red) for better orientation. (d) Reconstructed height profiles and membrane fluidity of cell membra. The right panels show the histogram of GP values with different height thresholds (n = 10). The results indicate that the region of close contact (0 < h nm < 40) is a low-ordered membrane with high fluidity. (e) Zeta potential analysis of different insect cell lines, C6/36: albopictus, S2: Drosophila, SES-MaBr-3: *Mamestra brassicae*. Representative reflectance images of SES-MaBr-3 are shown in the right panels. The scar bar corresponds to 10 μm.

After (right) of Figure S3: Quantitative evaluation of topological and membrane fluidity profiles of insect cell adhesion. (Extended data for Figure 3). (a) Preparation of glass substrates with different charges by silane coupling. (b) Time course of representative IRM images and cropped contact area (h ≤ 40 nm) of Sf21 cells after treatment of 1.5 μM lovastatin (inhibitor for cholesterol synthesis) for 3 days. (c) Statistical analysis of the corresponding contact area during incubation (n = 5). The error bar represents the standard deviation. (d) Representative histograms of GP(Generalized polarization) before and after treatment of 10 mM MβCD. Region of tight contact region (h = 0 – 40 nm) and detached region (h = 40 – 80 nm) is displayed in red and blue color respectively. (e) Zeta potential analysis of different insect cell lines, C6/36: albopictus, S2: Drosophila, SES-MaBr-3: *Mamestra brassicae*. Representative reflectance images of SES-MaBr-3 are shown in the right panels. The scar bar corresponds to 10 μm. (f) Representative IRM and fluorescence images of Sf21 cells after adding 10 μM bombykal. (f) Here, Sf21 cells expressing BmOR3, which specifically responds to bombykal, were used, and the fluorescence from GCaMP6 was monitored. Detailed protocol for establishing BmOR3 positive Sf21 cell lines was described in our previous papers[12].

Revised Table Of Contents

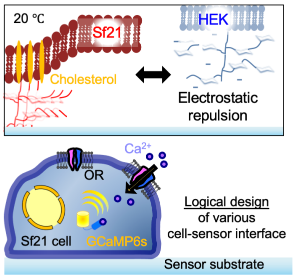

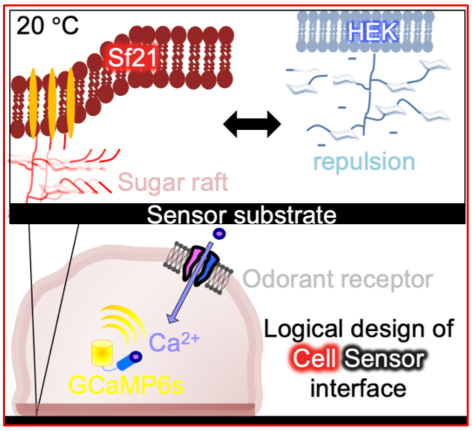

Before (left) and After (right)of Table of contents
